# Evaluation of Oxfendazole, Praziquantel and Albendazole against Cystic Echinococcosis: A Randomized Clinical Trial in Naturally Infected Sheep

**DOI:** 10.1371/journal.pntd.0000616

**Published:** 2010-02-23

**Authors:** Cesar M. Gavidia, Armando E. Gonzalez, Eduardo A. Barron, Berenice Ninaquispe, Monica Llamosas, Manuela R. Verastegui, Colin Robinson, Robert H. Gilman

**Affiliations:** 1 Veterinary School, Universidad Nacional Mayor de San Marcos, San Borja, Lima, Peru; 2 Department of Microbiology, School of Sciences, Universidad Peruana Cayetano Heredia, Lima, Peru; 3 Tufts University School of Medicine, Boston, Massachusetts, United States of America; 4 Bloomberg School of Public Health, The Johns Hopkins University, Baltimore, Maryland, United States of America; Universidad Nacional Autónoma de México, Mexico

## Abstract

**Background:**

Cystic Echinococosis (CE) is a zoonotic disease caused by larval stage *Echinococcus granulosus*. We determined the effects of high dose of Oxfendazole (OXF), combination Oxfendazole/Praziquantel (PZQ), and combination Albendazole (ABZ)/Praziquantel against CE in sheep.

**Methodology/Principal Findings:**

A randomized placebo-controlled trial was carried out on 118 randomly selected ewes. They were randomly assigned to one of the following groups: 1) placebo; 2) OXF 60 mg/Kg of body weight (BW) weekly for four weeks; 3) ABZ 30 mg/Kg BW + PZQ 40 mg/Kg BW weekly for 6 weeks, and 4) OXF 30 mg/Kg BW+ PZQ 40 mg/Kg BW biweekly for 3 administrations (6 weeks). Percent protoscolex (PSC) viability was evaluated using a 0.1% aqueous eosin vital stain for each cyst. “Noninfective” sheep were those that had no viable PSCs; “low-medium infective” were those that had 1% to 60% PSC viability; and “high infective” were those with more than 60% PSC viability. We evaluated 92 of the 118 sheep. ABZ/PZQ led the lowest PSC viability for lung cysts (12.7%), while OXF/PZQ did so for liver cysts (13.5%). The percentage of either “noninfective” or “low-medium infective” sheep was 90%, 93.8% and 88.9% for OXF, ABZ/PZQ and OXF/PZQ group as compared to 50% “noninfective” or “low-medium infective” for placebo. After performing all necropsies, CE prevalence in the flock of sheep was 95.7% (88/92) with a total number of 1094 cysts (12.4 cysts/animal). On average, the two-drug-combination groups resulted pulmonary cysts that were 6 mm smaller and hepatic cysts that were 4.2 mm smaller than placebo (p<0.05).

**Conclusions/Significance:**

We demonstrate that Oxfendazole at 60 mg, combination Oxfendazole/Praziquantel and combination Albendazole/Praziquantel are successful schemas that can be added to control measures in animals and merits further study for the treatment of animal CE. Further investigations on different schedules of monotherapy or combined chemotherapy are needed, as well as studies to evaluate the safety and efficacy of Oxfendazole in humans.

## Author Summary

Cystic Echinococcosis (CE) is a near-cosmopolitan parasitic zoonosis that causes economic losses in many regions of the world. This parasitic infection can be regarded as an emerging or re-emerging disease causing considerable losses in livestock production. CE is produced by the larval cystic stage (hydatid) of the dog parasite *Echinococcus granulosus*. After infective eggs are ingested, cysts develop mainly in lungs and liver of humans and animals (sheep, cattle, pigs, horses, etc). Infected people may require surgery and/or Albendazole-based chemotherapy. In this study, we evaluated the effects of Oxfendazole alone (an antiparasitic drug used in animals), Oxfendazole plus Praziquantel, and Albendazole plus Praziquantel against hydatid cysts in sheep over 4 to 6 weeks of treatment. All of the treatments in this study were efficacious in killing the larval stages and, therefore, in minimizing the risk of a dog acquiring new infections (taenias). These treatment schemes can be added to control measures in animals and eventually could be used for the treatment of human infection. Further investigations on different schedules of monotherapy or combined chemotherapy are needed, as well as studies to evaluate the safety and efficacy of Oxfendazole in humans.

## Introduction

Cystic Echinococosis (CE) is a zoonotic disease caused by larval stage or metacestode of *Echinococcus granulosus*. It is recognized as a major economic and public health problem in many areas worldwide [1]–[3]. The intermediate hosts include sheep, goats, cattle, horses, swine, and other wild ruminants; humans can be considered as accidental hosts. The larval stage frequently inhabits the liver and lungs both in humans and animals [4]. The hydatid cyst then expands and can contain hundreds or thousands of PSCs that can develop into the adult tapeworm upon ingestion by the definitive host, typically dogs, wolves, and wild canids [5],[6].

When surgery is not an option as first-line treatment for humans, antiparasitic drugs are used in the treatment of CE [5],[7],[8]. Through animal trials, mebendazole and albendazole (ABZ) have been shown effective against both *E. granulosus* and *E. multilocularis* larval stages [9]-[15]. The current chemotherapy for human CE is 2-4 cycles of ABZ for 30 to 60 days with 15-days breaks between cycles [5],[14]. A review of ABZ uses found that the proportion of cured cysts ranged from 11.8% to 35.2%, while between 22.4% to 50.0% remained unchanged [14],[15]. ABZ treatment is associated with anomalies in hepatic function, leukopenia and alopecia among other side effects [14]–[16]. Despite the questionable efficacy and high cost, ABZ remains the best treatment option for inoperable human cases and is the drug of choice for perioperative prophylaxis [1] due to the lack of alternative drugs against hydatid cysts.

ABZ, along with levamisol and ivermectin, is also one of the most used antiparasitic drug in sheep, goats, cattle, horses and pigs. It is recommended against a variety of nematodes as well as trematodes [17]. Although ABZ is still regularly used in Australia, Africa, Europe, North and South America, reports of nematode resistance have emanated from those areas [18]. It has been shown that ABZ is not detected in the plasma of treated sheep at any time after administration, nevertheless, its active metabolites, albendazole sulphoxide and albendazole sulphone are detectable for about 48 hr after administration. Albendazole sulphoxide is the most important antihelmintic active metabolic product recovered from the bloodstream after treatment of sheep with ABZ [19]. The antiparasitic activity of the active metabolite depends largely not only on its affinity for parasite β-tubulin, but also on its ability to reach high and sustained concentrations at the site of parasite location [20].

Oxfendazole (OXF), another benzimidazole like ABZ, has broad-spectrum activity against larval stages of gastrointestinal roundworms, tapeworms, and lungworms in many animal species [21]. OXF has not yet been approved for human use making an enormous disadvantage compared with ABZ. However, while the main ABZ active metabolite (albendazole sulfoxide) can be found in plasma for 60 hours post-treatment, OXF (active drug) can be found in plasma up to 144 hours post-treament in a comparative plasma availability study in sheep [22]. Animal studies have demonstrated that administering OXF in doses of 30 mg/Kg for 11 consecutive weeks (sheep) [23],[24] or biweekly for 4 weeks (goats) [25] results in efficacies greater than 90% against cystic echinococcosis. OXF has also been evaluated in combination with Nitazoxanide in sheep, and it was shown that most of the effect against hydatid cysts was attributable to the OXF [23].

Although OXF has been evaluated in sheep and goats, there are no clinical trials in sheep to determine the efficacy of higher dosages of OXF in shorter periods of treatment. Also, there is not information concerning the efficacy of combination with other antiparasitic drugs rather than Nitazoxanide. We investigated in a randomized placebo-controlled trial the effects of OXF alone as well as the combinations of OXF/PZQ and ABZ/PZQ against CE in naturally-infected sheep.

## Methods

### Study design

We performed a randomized placebo-controlled trial and selected 118 ewes, which were then assigned to one of the three treatment groups or a placebo group. Sheep were identified and treated under different dosage schedules. The ewes were then sacrificed 4 to 8 weeks after the last treatment for collection and evaluation of lungs and livers. We counted the number of cysts in each organ, and observed their morphological characteristics, fertility status, and PSC viability to estimate the treatment efficacy. The study was approved by the ethical review boards of the Universidad Nacional Mayor de San Marcos, School of Veterinary Medicine. We tested the hypothesis that OXF in higher doses or combined with PZQ would reduce the PSC viability and the cyst size in both lung and liver hydatid cysts.

### Animals

Sheep were obtained from the Tupac Amaru, ranching cooperative located in the Peruvian Central Highlands. This area has a high prevalence of CE in sheep (87%), and humans (9.1%) as previously reported [26]. Sheep were selected and assigned to groups in two steps. First, a total of 118 Junin-breed ewes with eight-tooth erupted (28 to 48 months of age) were randomly selected by applying systematic random sampling procedure in a corral from a flock of 700 sheep already determined for culling by the cooperative. Animals that were clinically sick and unable to move and feed by themselves were excluded before applying the systematic randomization. Each selected sheep was identified using numbered ear tags at the beginning of the study. Next, having a list with those selected and numbered sheep, a simple random procedure to allocate the animals into four groups was applied. These animals were maintained in the same corral with the rest of the flock under identical food and water availability. Animal conditions were monitored prior to and throughout the experimental period.

### Treatment groups

Sheep were randomly assigned into four groups as follows: 1) placebo; 2) OXF 60 mg/Kg of body weight (BW) weekly for four weeks; 3) ABZ 30 mg/Kg BW + PZQ 40 mg/Kg BW weekly for 6 weeks, and 4) OXF 30 mg/Kg BW+ PZQ 40 mg/Kg BW biweekly for 3 doses (6 weeks). The placebo group received a mix of flour with water and was handled under similar conditions for 6 weeks to cause the same stress as the other groups. For the combination groups, we gave the treatments separately; the drugs were kept in their original bottles and they were not mixed together.

All drugs were given using an oral drench via a Hauptner automatic oral dose syringe (Nasco, Modesto, CA) and were administered from a bottle suspension of oxfendazole (Synanthic 22.5% solution), or Albendazole (Vetalben, 15%) or Praziquantel (Saniquantrel, 10%). The cost of each dose of OXF for an average weight sheep (45 Kg) was US$0.36 at 30 mg/kg BW and US$0.72 at 60 mg/Kg BW. The combination of ABZ/PZQ as a single dose costs US$0.64 for an average weight sheep, and the combination of OXF/PZQ as a single dose costs US$0.88. Animals were checked 3–5 minutes after administration to ensure that they had swallowed the full dose of medication.

### Sample size calculation

The sample size was estimated using the formula for testing differences of two proportions, using a two-sided test with significance level α = 0.05 and power of 80%, with equal size for groups [27]. Based on previous findings of Oxfendazole efficacy in sheep of 90% (dead protoscoleces), and the 30% dead protoscoleces for the placebo group [24],[25], the estimated sample size was 10 for each study group. To compensate for some loss to follow up, we enrolled between 25 to 32 sheep per group.

### Necropsy procedures and cyst evaluation

Animals were slaughtered 4 to 8 weeks after their last treatment in the official abattoir of the Tupac Amaru Cooperative. Sheep were assigned correlative numbers in the slaughterhouse in order to blind lung and liver cyst evaluations to all study personnel. These codes were later matched with the originals (numbered ear tag) for statistical analysis. Only one person (designated by the principal investigator) was responsible for numbering the sheep and later matching the codes.

All cysts from both livers and lungs were counted and registered on individual sheets. Up to ten cysts per organ were measured (diameter) and classified according to their macroscopic appearance and hydatid fluid characteristics. For macroscopic evaluation, we used the description reported by Dueger and Gilman [24] of normal, calcified and degenerated cysts. Normal cysts have a white inner layer filled with clear liquid; presence of a calcareous deposit in the inner wall denoted calcified cysts; and degenerated cysts had blackened walls filled with turbid or purulent liquid and degraded membranes.

Fertility was assessed for the fluid of each evaluated hydatid cyst. Hydatid fluid was placed in a 50 ml Falcon tube and gravity sedimented for 30 minutes. Sediment of each cyst was observed under a light microscope to look for PSCs. Cysts with PSCs were defined as fertile, and cysts without PSCs were considered infertile and not used for viability analysis. The cyst fertility percentage was calculated for each group (fertile cysts/total cysts). Viability was performed using a 0.1% aqueous eosin vital stain [28]. Dead, nonviable PSCs stain red while those that are viable exclude the dye and remain clear. The percentage of live PSCs divided by the total PSCs (live PSC/total PSC) was used to calculate the percent PSC viability for each cyst for both lung and liver.

### Data analysis

All data analysis was performed according to a pre-established analysis plan. We defined sheep infection status based on the studies of Dueger and Gilman with some modifications [24]. When all the sheep's fertile cysts had 0% PSC viability, they were called “noninfective”; sheep with all fertile cysts having 1% to 60% PSC viability were considered as “low-medium infective”; and sheep with all fertile cysts with more than 60% PSC viability were called “highly infective”. It has been previously shown that without treatment the proportion of sheep with viable cysts is generally higher than 60% [29]–[31], thus we used that cut-off to create these categories of infective animals. For the purposes of this study, the primary endpoint with respect to efficacy of any treated group was the noninfective and/or low-medium infective sheep rates as measured by the percentage of PSC viability.

Additionaly, the Kruskall-Wallis test was used to estimate the difference in the number of cysts between treatment groups. Analysis of Variance (ANOVA) was performed for cyst diameter and Bonferroni multiple-comparison test was used to establish the pair-group difference. The chi-square test was used to assess the association of fertility and degenerated cysts (calcified and purulent cysts included) with treatment groups. Logistic regression models were used to estimate the odds of having degenerated or fertile cysts; treatment and organ were the independent variables with cluster in animal codes. For all the analyses, the significant differences were set to less than 0.05. Percentages are presented with their 95% confidence intervals.

## Results

**Figure 1:**
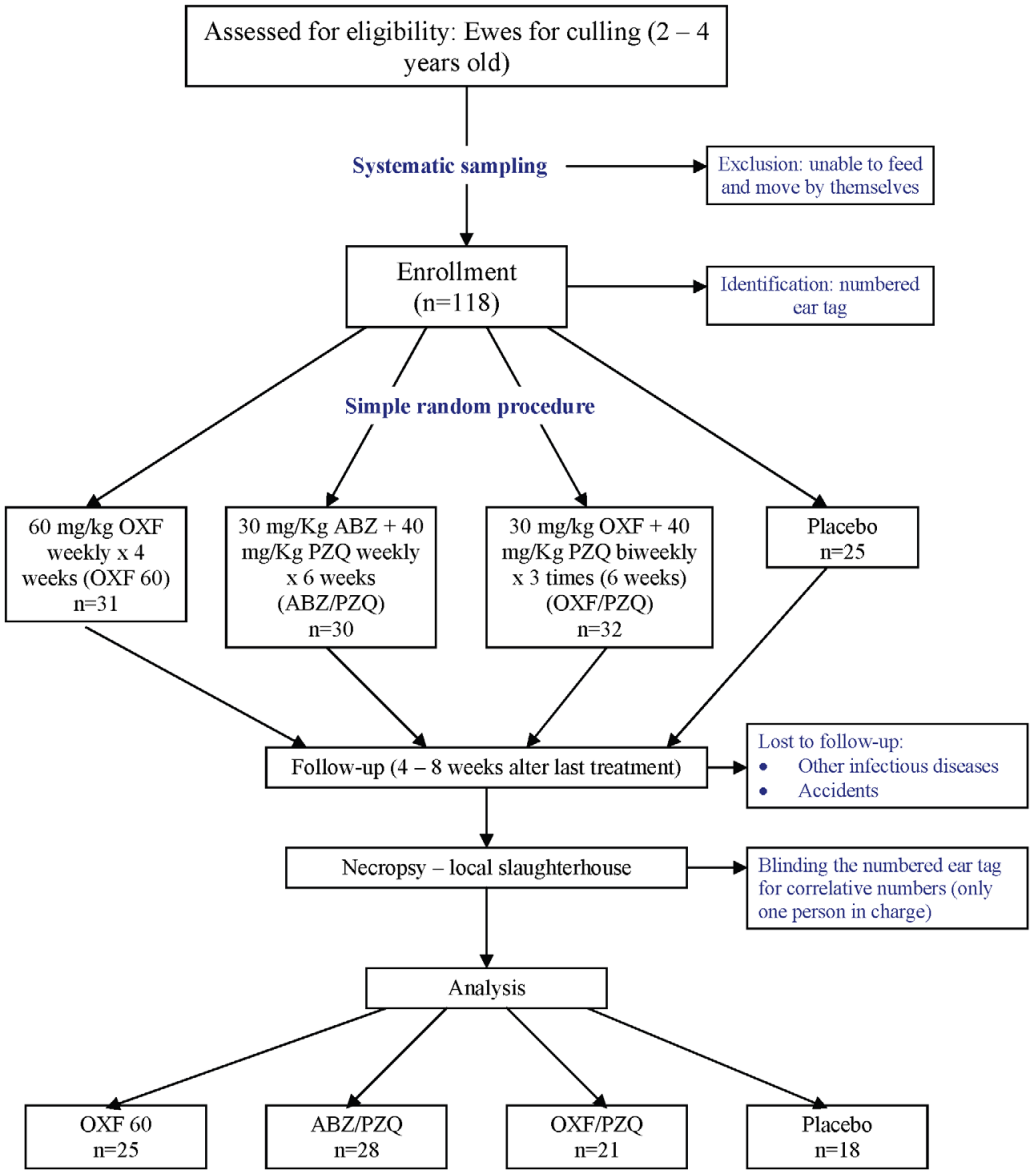
Randomized clinical trial of infected sheep to evaluate Oxfendazole, praziquantel and albendazole against cystic echinococcosis. The flowchart is presenting the phases of the clinical trial. The first part (starting on top) is showing the process and criteria of sampling and assigning sheep to different treatment groups. The second part (in the middle) is presenting the group definitions, sample sizes and follow up of the animals. The third part (on the bottom) is showing the last activities and number of animals per groups that were necropsied at the end of the clinical trial.

This study recruited a total of 118 Junin bred eight-tooth sheep (28–48 month-old ewes) during the first week of June 2006. The average mean weight was statistically similar among the groups at the beginning of the study as determined by the Analysis of Variance test. From the original 118 animals, only 92 were examined in the slaughterhouse. Twenty-six sheep were lost or had died due to non-treatment related causes such as other infectious diseases and accidents. These 26 animals were equally distributed among the groups as seen in Table 1. All animals were similar in weight at the beginning of the study (Figure 1) and no side effects were observed during the period of this trial. Animals were followed-up 4 to 8 weeks after the last treatment.

**Table 1:** Number of sheep at the pre- and post-treatment, and number of lost and cystic echinococcosis-negative animals in the trial.

Group	Pre-treatment^a^	Post-treatment^b^	Lost (%)^c^	Negatives
PLACEBO	25	18	7 (28.0)	1
OXF 60 mg	31	25	6 (19.4)	0
ABZ30 mg+PZQ40 mg	30	28	2 (6.7)	0
OXF30 mg+PZQ40 mg	32	21	11 (34.4)	3
TOTAL	118	92	26 (22.0)	4

a Pre-treatment: number of sheep that were treated at the beginning of this trial.

b Post-treatment: number of sheep that were evaluated and the end of this trial at the slaughterhouse. Negative sheep are counted in this column.

c Chi-square test showed no difference in the number of lost sheep between the groups.

After all the necropsies were performed the prevalence of CE could be determined in the sheep. There were a total of 4 negative sheep (no cysts either in lung or liver), yielding a CE prevalence of 95.7% (88/92). The percentage of dual infection (lung and liver) was 84.8% (78/92). In the 88 positive sheep, there was a total of 1094 cysts (mean = 12.4 cysts/animal), of which 592 (54.1%) were pulmonary and 502 (45.9%) were hepatic, giving a lung-to-liver ratio of 1.2∶1. As seen in Table 2, there was no difference in the number of cysts for either organ, lung or liver, between the treatment and placebo groups. From those 1094 cysts in the whole group, we only were able to evaluate 845 (441 pulmonary, and 404 hepatic cysts) due to logistic and lab facility constraints.

**Table 2:** Total number of hydatid cysts and number of evaluated cysts for lung and liver by treatment group.

GROUP	LUNG*		LIVER*		TOTAL	
	Total	Evaluated	Total	Evaluated	Total	Evaluated
PLACEBO	112	87	107	87	219	174
OXF 60 mg	188	128	167	130	355	258
ABZ30 mg+PZQ40 mg	194	147	137	116	331	263
OXF30 mg+PZQ40 mg	98	79	91	71	189	150
TOTAL	502	441	502	404	1094	845

*: Kruskall-Wallis test found no statistical difference in the total number of cysts between the groups for both lung or liver.

There was a statistically significant difference in the diameter for both pulmonary and liver cysts as estimated by ANOVA (p<0.05). The two drug-combination groups had significantly smaller pulmonary cysts than placebo (means 18.9 mm for ABZ/PZQ, 17.9 mm for OXF/PZQ vs 24.4 mm for placebo) (p<0.05) (Table 3). For liver cysts, all of the treatment groups were similar to each other and all had smaller hepatic cysts than the placebo group as demonstrated by Bonferroni test (p<0.05). Hepatic cysts from OXF 60 mg, ABZ/PZQ and OXF/PZQ were 11.1 mm, 7.7 mm and 13.6 mm smaller than placebo group, respectively (Table 3).

**Table 3:** Average diameter and standard deviation (SD) of lung and liver cysts by treatment group.

GROUP	LUNG		LIVER	
	Mean ± S.D	n	Mean ± S.D	n
PLACEBO^a^	24.4±17.6^c^	87	27.8±21.8^e^	87
OXF 60 mg^b^	20.3±13.2^c^	128	16.7±12.0^f^	130
ABZ30 mg+PZQ40 mg^b^	18.9±14.4^d^	147	14.2±11.5^f^	116
OXF30 mg+PZQ40 mg^a^	17.9±14.5^d^	79	20.1±19.8^f^	71

a Mean diameter difference was not statistically significant between lung and liver cysts evaluated by two-sample T-test for placebo and OXF/PZQ groups.

b Liver cysts were statistically smaller than lung cysts for OXF 60 mg and ABZ/PZQ groups (p<0.05) as determined by two-sample t test.

c Placebo group and OXF 60 mg had equal lung cyst diameters.

d ABZ/PZQ and OXF/PZQ had statistically significant smaller lung cysts than the placebo group (p<0.05), but they were similar in size to OXF 60 mg.

e,f All treatment groups had statistically significant smaller liver cysts than the placebo group (p<0.05), but there were no statistically significant differences between treatment groups.

Logistic regression analysis demonstrated that the odds of having fertile cysts was similar between the treatment groups and between the organs (lung and liver). Similarly, there was no difference on the odds of having degenerated cysts between the treatment groups and placebo. However, the odds of having degenerated cysts was 70% higher in livers as compared to lungs after controlling for treatment group (OR = 1.7, 95% CI: 1.1–2.7, p = 0.02) (Table 4).

**Table 4:** Odds ratio of cyst fertility and cyst type (normal and degenerated) according to treatment group and organ.

Dependent variable	Group	OR	95% CI	p
Cyst fertility^a^	Placebo	1.0	-	-
	OXF 60 mg	0.5	0.2–1.5	0.25
	ABZ/PZQ	0.6	0.2–1.6	0.32
	OXF/PZQ	0.6	0.2–2.2	0.43
	Lung	1.0	-	-
	Liver	0.8	0.6–1.1	0.23
Cyst type^b^	Placebo	1.0	-	-
	OXF 60 mg	1.7	0.6–4.5	0.28
	ABZ/PZQ	2.0	0.8–5.4	0.16
	OXF/PZQ	2.1	0.5–8.6	0.29
	Lung	1.0	-	-
	Liver	1.7	1.1–2.7^c^	0.02

a No difference was found for treatment groups and organ for cyst fertility.

b No difference was found for treatment groups for cyst type. Cyst type was analyzed as normal and degenerated (calcified and purulent cysts).

c The odds of having degenerated liver cysts was 70% higher than lung cysts after controlling for treatment groups, statistically significant at p = 0.02.

All of the treatment groups had, on average, significantly lower PSC viability than the placebo group for lung and liver cysts as shown in Table 5 (p<0.05). Although treated groups had PSC viability statistically similar each other for both lung and liver, the combination of ABZ/PZQ exhibited the lowest PSC viability for lung cysts (12.7%), while OXF/PZQ did for liver cysts (13.5%) (Table 5).

**Table 5:** Grouped-average PSC viability by organ and treatment group.

Group	% of PSC viability by organ	
	Lung	Liver
PLACEBO	58.4^a^	45.9^c^
OXF 60 mg	23.8^b^	15.1^d^
ABZ30 mg+PZQ40 mg	12.7^b^	18.8^d^
OXF30 mg+PZQ40 mg	15.6^b^	13.5^d^

a,b Placebo group for lung cysts was statistically larger than any of the treatment groups (p<0.05), but all treated groups were similar to each other.

c,d Placebo group for liver cysts was statistically different from each of the three treatment groups (p<0.05), but the treated groups were similar to each other.

Taking only those sheep with fertile cysts, the proportion of “noninfective” sheep was 8.3%, 30%, 25% and 33.3% for placebo, OXF60 mg, ABZ/PZQ and OXF/PZQ groups, respectively. Additionally, the percentage of either “noninfective” or “low-medium infective” sheep was only 50% for placebo, while these rates were 90%, 93.8% and 88.9% for OXF 60 mg, ABZ/PZQ and OXF/PZQ groups, respectively. In contrast, the proportion of “highly infective” sheep was near or less than 10% for treated groups as compared to 50% for placebo one (Table 6). Also, the combined-drug groups had a lower proportion of fertile cysts (ABZ/PZQ–16/28, or 57.1%; OXF/PZQ–9/21, or 42.9%) than placebo (12/18, or 66.7%) and OXF 60 mg (16/23, or 70%) though no statistically difference was demonstrated.

**Table 6:** The effect of Oxfendazole, ABZ plus PZQ and OXF plus PZQ on sheep cystic echinococcosis as measured by PSC viability.

Group (n)	N° of sheep with fertile cysts	Noninfective^b^	Low-medium infective^c^	Highly infective^d^
PLACEBO (18)	12	1 (8.3%)	5 (41.7%)	6 (50.0%)
OXF 60 mg (25)	20	6 (30.0%)	12 (60.0%)	2 (10.0%)
ABZ30 mg+PZQ40 mg (23)	16	4 (25.0%)	11 (68.8%)	1 (6.3%)
OXF30 mg+PZQ40 mg (28)	9	3 (33.3%)	5 (55.6%)	1 (11.1%)

a No difference was found in the number of sheep between the groups and the final condition (noninfective, low-medium infective, highly infective) by using the chi-square test.

b,c No difference was demonstrated between any of the treated groups and the placebo group by means of two-sample test of proportion. However, when adding noninfective and low-medium infective sheep, each of the treatment groups was significantly higher than placebo group (p<0.05).

d OXF 60 mg and ABZ/PZQ had statistically significant lower proportions of highly infective sheep than did the placebo group (p<0.05) by two-sample test of proportion. OXF/PZQ group was close to the borderline as compared to placebo (p = 0.06).

## Discussion

This study demonstrated that Oxfendazole at 60 mg/Kg, or the combinations Oxfendazole/Praziquantel and Albendazole/Praziquantel act against Cystic Echinoccosis in naturally-infected sheep. All of the treatment schedules were able to reduce both hepatic and pulmonary cyst size compared to placebo. Protoscolex viability was lower statistically for all of the treatment schedules than placebo animals for both lung and liver cysts. In the OXF alone, ABZ/PZQ and OXF/PZQ groups, 90%, 93.8% and 88.9% of sheep, respectively, were either “noninfective” or “low-medium infective” following treatment, compared to 50% noninfective or low-medium infective animals in the placebo group.

Oxfendazole was previously evaluated using different approaches and dosages in both sheep and goats. The longest trial used OXF at 30 mg/Kg for eleven consecutive weeks [24] while the other two used OXF at 30 mg/Kg twice a week for four weeks [25],[32]; all of them achieved high efficacy against CE (∼90%). In our trial, we increased the OXF dose (60 mg/Kg), and for the first time evaluated the combination of OXF/PZQ. We obtained similar results to those previous studies with efficacies of 90% and 88.9% for OXF 60 mg and OXF/PZQ, respectively.

Furthermore, we applied a more practical schedule using a less intensive regimen (once weekly or biweekly), reducing not only the treatment period but also minimizing the unnecessary animal handling as compared to the three previous trials. Long treatment periods in sheep are not only impractical but also costly in developing countries where sheep are raised extensively in large areas of land to minimize outlay and labor.

In terms of OXF phamacokynetics, this drug is stored in the rumen of ruminants and it is detectable for up to seven days after administration [22],[33]. Therefore, we found no need to dose the animals twice weekly as it has been done before [25]; instead we dosed the animals once weekly and doubled the dose. Unfortunately, there are no reports that describe the toxic level of OXF in sheep, so we could not investigate higher doses of this drug.

In spite of variable ABZ effectiveness, the current chemotherapy for human CE is 2–4 cycles of ABZ for 30 to 60 days with 15-days breaks between cycles [5],[14]. A review of ABZ uses found that the proportion of cured cysts ranged from 11.8% to 35.2%, while between 22.4% to 50.0% remained unchanged [14]. A prospective study carried out over 300 liver cysts, 42 abdominal cysts and 56 lung cysts found that 77.9% of the cysts, irrespective of their localization, showed degenerative modifications after patients received 10–12 mg/kg/day of ABZ without intervals for 3 months. Additionally, 30.9% recurred and 50% relapsed more than once. Overall success was 49.3% while failure resulted in 31.5% of the patients [34].Although the cure rates of our study were not the ultimate goal of treatment, they ranged between 25.0% to 33.3%, lower than those reported by Teggi et al [34] with ABZ, but in a shorter regimen without any clinically detectable side effect. Furthermore, OXF alone or the combinations OXF/PZQ and ABZ/PZQ were able to significantly decrease the cyst size as well as to reduce the number of viable PSCs using a shorter regimen than the current use of ABZ. However, this study's conclusions are limited to ruminants for two reasons. First, OXF is not licensed for human use and it must wait until it is proven to be safety and efficacious in persons. Second, OXF blood levels, phamacodynamic and phamacokinetics may be different in monogastrics. It is, nevertheless, worthwhile to point out the unknown potential of OXF or combinations in animal intermediate hosts to spark the interest of other investigators.

Regarding drug combinations, the recommended dose of ABZ for human CE is 10 mg/Kg to 15 mg/Kg while PZQ is recommended at 25 mg/Kg to 40 mg/Kg [5]. However, a recent review concluded that one of the major unanswered clinical questions is whether the combination ABZ/PZQ is superior to ABZ alone when both effectiveness and drug toxictiy are taken into account [35]. In mice, PZQ and ABZ as combined therapy against experimental hydatid disease was 100% effective as chemoprophylactic treatment but no significant difference was found to cure secondary experimental cystic echinococcosis [36]. In our study, the two combination therapies (ABZ/PZQ and OXF/PZQ) were efficacious in curing or decreasing PSC viability in already-infected sheep. However, we must point out that the combination of OXF with PZQ might lack the necessary evidence and the real mechanism against CE would require further investigations. PZQ has the ability to increase the active metabolite of ABZ (ABZ sulphoxide) up to 4.5-fold [5]; however, no information is available about the effect of PZQ over OXF metabolites. In spite of the high doses used in this trial, no side effects were observed in any of the animals suggesting that all of the therapeutic schemes were safe at least for sheep.

Our trial demonstrated that both pulmonary and hepatic cysts from treated groups were reduced in size as compared to those from the placebo. Although that difference might not be clinically relevant (approximately 10 mm in diameter for hepatic cysts), cyst reduction is one of the indicators of successful treatment as evidenced in chemotherapy studies in humans. For instance, a study evaluating the timing and degenerative changes in the cysts after patients received benzimidazole treatment demonstrated that abdominal cysts (<5 cm diameter) became smaller after 3.3–9.3 months [37]. We furthermore showed that the decreased cyst diameter can be observed in a relatively short period of time (4–8 weeks). Although our treatment options are not yet applicable to humans, future clinical trials in humans or animals might take our results as a reference.

Another interesting application of chemotherapy may be as part of a control program. For instance, PZQ treatment of dogs will stop new infections in sheep but will not affect the reservoir of cysts present in already-infected sheep [38]. On the other hand, sheep cystic echinococcosis can be prevented by using a highly effective vaccine [39], but to date it has not been used in any national control program due to both cost and logistics. Furthermore, infection acquired before sheep vaccination can still be transmitted to dogs, and it can take up to 15 years before all cyst reservoirs (infected sheep) disappear from a specific herd [38]. Therefore, protoscolicidal drugs, such as OXF or the combinations of ABZ/PZQ and OXF/PZQ, might be additional strategies to for control programs. Efficacious, inexpensive, and practical and expeditious treatments may reduce the number of viable PSCs. These “noninfective” offal from infected sheep will be less likely to yield new taenias, decreasing the number of dogs infected and/or the number of worms found in susceptible dogs.

Although we did not find any significant difference in the number of cyst between the groups, it is very likely that the number and size of the cysts may have been different between groups from the beginning of the experiment. Both characteristics - number and size - are time-dependent, thus sheep aged 2 years would have had fewer and smaller cysts than sheep aged 4 years or older, even if randomization was appropriately applied in field. We were not be able to differentiate the exact age in these group of sheep since we used tooth eruption as a surrogate of the age. All selected animals had eight-tooth already erupted, meaning they were all 28 to 48 months old. This limitation may have biased our results, probably by overestimating the drugs' efficacies. As shown by Torgerson et al in a mathematical simulation [40], there is high probability of a substantial difference in worm burdens if allocations are undertaken randomly at the beginning of any clinical trial. It was demonstrated that even with identical initial experimental infections with *Haemonchus contortus* in sheep, fewer worms established in one of the groups (Moxidectin) as compared to the other groups (Ivermectin, Mebendazole, and control) before the onset of treatment. Thus, the apparent parasitic reduction in our trial could be overestimated since some sheep may have had fewer cysts initially; hence drug efficacy may also be overestimmated. It is already known that the distribution of macroparasites over their host population is highly aggregated in naturally infected animals [41]. We would, therefore, suggest that more exact age determination be used and that sheep should be assigned to groups on the basis of age-matching in future animal clinical trials.

One of the limitations of our study is the short time between final treatment dose and animal sacrifice. A trial evaluating PZQ and ABZ in sheep waited up to 7 months before slaughtering the animals to allow any residual live parasites to recover [42]. Since we evaluated the animals only 4 to 8 weeks after treatment, we do not know whether the effect of the drugs might have been prolonged or whether surviving parasites (viable PSCs) would re-activate cysts in the future. Additionally, the absence of living PSCs in a cyst does not mean the cyst is dead or inactive, merely that the germinal layer is not producing PSCs at the time. For a cyst to be non-viable, one would need to show that the totality of the cyst is incapable of re-establishing the infection for a period of time, or to demonstrate histologically the complete destruction and degeneration of the germinal layer. Unfortunately, this study was not able to produce that information and thus we refer to “PSC viability” instead of “cyst viability” to evaluate the efficacy of the drugs. Another limitation of our study is the lack of information of pharmacokinetic, pharmacodynamic, and toxicity data of OXF when combined with other antiparasitic drugs in other animal species. Thus we might not have sufficient background to select the appropriate dose of OXF when combining with PZQ.

In summary, this trial demonstrates that Oxfendazole at 60 mg/kg and the combined therapies of Oxfendazole + Praziquantel (30 mg/Kg + 40 mg/Kg) and Albendazole + Praziquantel (30 mg/Kg + 40 mg/Kg) are successful agents that can be added to current control measures to interrupt the transmission of *Echinococcus granulosus*. It also demonstrates the potential of these antiparasitic drugs to be used in the treatment of Cystic Echinococcosis in a relatively short period of time, reducing the cost of the therapy. Further investigations of varying doses of monotherapy and combined chemotherapy are needed, as well as evaluation of the efficacy and safety of OXF in humans.

## Supporting Information

Alternative Language Abstract S1 Spanish translation of the abstract by CMG.
(0.03 MB DOC)

Checklist S1 CONSORT checklist.
(0.06 MB DOC)

Protocol S1 Treatment of Cystic Echinococcosis in sheep.
(0.12 MB DOC)
