# Supplementary material for: Evaluation of Oxfendazole, Praziquantel and Albendazole against Cystic Echinococcosis: A Randomized Clinical Trial in Naturally Infected Sheep
Source: PLoS Negl Trop Dis. 2010 Feb 23;4(2):e616. doi: 10.1371/journal.pntd.0000616 (PMC2826409; doi:10.1371/journal.pntd.0000616)
Supplement: Alternative Language Abstract S1 — Spanish translation of the abstract by CMG. (0.03 MB DOC) [file pntd.0000616.s001.doc]

**Evaluation of Oxfendazole, Praziquantel and Albendazole against Cystic Echinococcosis: randomized clinical trial in naturally infected sheep**

**ABSTRACT**

**Introducción**: La equinococosis quística (EQ) es una enfermedad zoonótica causada por el estadio larval del *Echinococcus granulosus*. Este estudio determinó el efecto de una dosis alta de Oxfendazole (OXF), la combinación de Oxfendazole/Praziquantel (PZQ) y la combinación Albendazole (ABZ)/Praziquantel contra EQ en ovinos.

**Metodología / Hallazgos principales**: Un ensayo clínico controlado se llevó a cabo con un total de 118 ovejas seleccionadas al azar. Los animales fueron asigandos aleatoriamente a uno de los siguientes grupos: 1) placebo; 2) OXF 60mg/Kg de peso vivo (PV) semanalmente por 4 semanas; 3) ABZ 30mg/Kg PV + PZQ 40mg/Kg PV semanalmente por 6 semanas, y 4) OXF 30mg/Kg PV+ PZQ 40mg/Kg PV cada dos semanas por 3 veces (6 semanas). El porcentaje de viabilidad de protoscolex (PSC) se evaluó usando eosina al 0.1% para cada quiste. Los ovinos “no infectivos” fueron aquellos que tuvieron una viabilidad de PSC de 0%; los que tuvieron una viabilidad entre 1% y 60% se denominaron “bajo-moderadamente infectivos”; y aquellos con viabilidad superior a 60% se llamaron “altamente infectivos”. Nosotros evaluamos 92 de los 118 ovinos. El grupo ABZ/PZQ tuvo la más baja viabilidad de PSC en quistes pulmonares (12.7%), mientras que el grupo OXF/PZQ lo tuvo para los quistes hepáticos (13.5%). El porcentaje de ovejas “no infectivas” o “bajo-moderadamente infectivas” fue 90%, 93.8% y 88.9% para los grupos OXF, ABZ/PZQ y OXF/PZQ comparado con un 50% “no infectivas” o “bajo-moderadamente infectivas” para el placebo. Después de realizar las necropsias, la prevalencia de EQ en el hato fue de 95.7% (88/92) con un total de 1094 quistess (12.4 quistes/animal). En promedio, los dos grupos de combinación de drogas tuvieron quistes pulmonares 6mm más pequeños que el placebo y 4.2mm más pequeños que el placebo para los quistes hepáticos (p<0.05).

**Conclusiones / Significancia**: Este estudio demuestra que Oxfendazole 60mg, la combinación de Oxfendazole/Praziquantel y la combinación Albendazole/Praziquantel son esquemas de tratamiento exitosos que pueden ser incluídos como medidas de control en animales y por lo tanto más estudios deberían ser realizados contra EQ en animales. Otras investigaciones con diferentes esquemas de tratamiento como monoterapia y combinaciones son necesarios, así como estudios para evaluar la seguridad y eficacia del Oxfendazole en humanos.
