# Supplementary material for: Evaluation of Oxfendazole, Praziquantel and Albendazole against Cystic Echinococcosis: A Randomized Clinical Trial in Naturally Infected Sheep
Source: PLoS Negl Trop Dis. 2010 Feb 23;4(2):e616. doi: 10.1371/journal.pntd.0000616 (PMC2826409; doi:10.1371/journal.pntd.0000616)
Supplement: Protocol S1 — Treatment of Cystic Echinococcosis in sheep. (0.12 MB DOC) [file pntd.0000616.s003.doc]

**TREATMENT OF CYSTIC ECHINOCOCCOSIS IN SHEEP**

# Site:

Cooperative Farm SAIS Tupac Amaru, located in the Central Peruvian Highlands. This is a Cystic Echinococcosis-endemic area.

# Beginning date:

May 31, 2006

# Trial name:

Randomized placebo-controlled trial of Oxfendazole, Praziquantel and Albendazole against Cystic Echinococcosis in naturally infected sheep.

# Hypothesis

High dose of Oxfendazole weekly for four weeks is efficacious against hydatid cysts in sheep.

# Objective:

To determine the effects of OXF administered as single drugs, as well as to compare the combinations of OXF plus PZQ and ALB plus PZQ against cystic echinococcosis in naturally infected sheep.

# Methodology

**Study design**

We will carry out a randomized placebo-controlled trial on 118 randomly selected ewes. They will be assigned to one of the three treatment groups or a placebo group. Sheep will be identified and treated under different dosage schedules. The ewes will be slaughtered 4 to 8 weeks after the last treatment for collection and evaluation of lungs and livers. We will count the number of cysts in each organ, and record their morphological characteristics, fertility status, and protoscolex viability to estimate the treatment efficacy. The study was approved by the ethical review boards of the Universidad Nacional Mayor de San Marcos, School of Veterinary Medicine.

**Sample size calculation**

The sample size was estimated using the formula for testing differences of two proportions, using a two-sided test with significance level α=0.05 and power of 80%, with equal size for groups [1]. Based on previous findings of Oxfendazole efficacy in sheep of 90% (dead protoscoleces), and the 30% dead protoscoleces for the placebo group [2, 3], the estimated sample size was calculated on 10 sheep per group.

$$n=\frac{\left[Z_{1-\alpha/2}\sqrt{2\overline{p}\overline{q}}+Z_{1-\beta}\sqrt{p_{1}q_{1}+p_{2}q_{2}}\right]^{2}}{\Delta^{2}}$$

**Animals**

Sheep will be obtained from the Tupac Amaru, a ranching cooperative located in the Peruvian Central Highlands which has a high prevalence of CE in sheep (87%), and humans (9.1%) as reported previously [4]. Animals will be randomly selected from a lot of 700 sheep already determined for slaughter by the cooperative. Systematic sampling will be applied as the animals enter in the corral. Animals that are unable to feed by themselves will be excluded during the selection process. Each selected sheep will be identified using numbered ear tags in the right ear at the beginning of the study.

During this process only one person will be responsible for registering the sheep information in a pre-designed sheet (Person 1). Three other personnel will be handled and identified the animals following the instructions of Person 1. The data will include: date, ear tag number, gender, and age (based on teeth eruption). The correspondent dose of specific drug will be calculated and written in the individual sheet.

**Treatment groups**

Random sampling process will be applied on the selected sheep using an Excel sheet. In order to easily identify the treatment groups, ewes will be marked with different colors by spraying a small spot on their back region. Therefore, sheep will be assigned to one of the following four groups: 1) placebo (red); 2) OXF 60mg/Kg of body weight (BW) weekly for four weeks (green); 3) ALB 30mg/Kg BW + PZQ 40mg/Kg BW weekly for 6 weeks (yellow), and 4) OXF 30mg/Kg BW+ PZQ 40mg/Kg BW biweekly for 3 doses (6 weeks) (grey). The placebo group will receive water and will be handled under similar conditions for 6 weeks to cause the same stress as the other groups.

All drugs will be given using an oral drench via a Hauptner automatic and gradable oral dose syringe (Nasco) and administered from a bottle suspension of oxfendazole (Synanthic 22.5%), Albendazole (Vetalben, 15%) or Praziquantel (Saniquantrel, 10%). Each bottle will have an independent syringe to avoid drug residuals being shared with different groups. The ewe will be restricted in a seated position, and a designated person will indicate the drug and quantity for each animal.

Animals will be monitored 3-5 minutes after administration to ensure that all of them have swallowed the full dose of their medication. Sheep will be maintained in the same corral during the study period and monitored every day for side effects (lethargy, dizziness, lack of appetite, etc).

In case of a sheep is lost or dies for other non-treatment related causes, it will be replaced. In this context, one animal will be randomly selected from the herd, identified and treated as appropriate. The replacement period will be the first 4 weeks of the study, and these animals will last the necessary time before slaughtering.

**Necropsy procedures and cyst evaluation**

Ewes will be slaughtered 4 to 8 weeks after their last treatment in the official abattoir of Tupac Amaru Cooperative. Animals will be assigned a correlative number by entering into the slaughterhouse by a designated person. Therefore, lungs and livers will be identified with those correlative numbers instead of the ear tag numbers for masking the cyst evaluation. Livers and lungs will be placed in a plastic bag with the correspondent number. These codes will be later matched with the originals after the statistical analysis has been performed.

All cysts from both livers and lungs will be counted and registered on individual sheets. Up to ten cysts per organ will be measured (length) and classified according to their macroscopic appearance and hydatid fluid characteristics. For macroscopic evaluation, we will follow the description reported by Dueger and Gilman (2001) [2] of normal, calcified and degenerated cysts. Normal cysts will have a white inner layer filled with clear liquid; presence of a calcareous deposit in the inner wall will denote calcified cysts; and degenerated cysts will have a blackened wall filled with turbid or purulent liquid and will have a degraded membrane.

Lung and liver examination

1. Look and touch carefully the lung/liver surface to count the total number of cysts.
2. Measure the length of 10 randomly selected cysts (if available) or all of them if less than 10, and register them on the sheep sheet for each organ.
3. Using a 10ml syringe and a needle of 20x1^1/2^, aspirate the fluid of each cyst by puncture the cyst wall.
   1. Put the fluid into a 50ml Falcon tube and identify it with the sheep code, date and organ.
   2. Record the fluid characteristics on the sheep sheet as clear, opaque, yellowish, purulent or bloody.
   3. Cut the cyst using a knife and record the cyst characteristics on each sheep sheet as follows
      1. Normal: white membranes with one or more inner chambers.
      2. Calcified: nodules, hardness, calcareous and/or chalk-like deposits
      3. Degenerated: dark membranes, purulent content
      4. Bloody
4. Chop and look for other pulmonary/hepatic parasites; register if any.
5. If no cysts are found in the lungs/liver, it will be registered as negative.
6. Cyst fertility will be assessed by examining the fluid of each cyst. Hydatid fluid will be placed in a 50ml Falcon tube and gravity sedimented for 30 minutes. Sediment of each cyst will be observed under a light microscope to look for protoscoleces (PSCs). Cysts with PSCs will be defined as fertile and registered as 1; otherwise they will be infertile (code 0) and not considered for viability analysis. The cyst fertility percentage will be calculated for each organ and group (number of fertile cysts / total cysts).
7. Protoscolex viability will be estimated using a 0.1% (1gr of Eosin with 1000ml of destiled water) aqueous eosin vital stain [5]. A ten-hole slide will be used to add 10ul of the hydatid fluid containing PSCs plus 10ul of the eosin solution on each hole. Dead, nonviable PSCs stain red while those that are viable exclude the dye and remain clear. The percentage of live PSCs divided by the total PSCs (live PSC / total PSC) will be used to calculate the percent protoscolex viability for each cyst for both lung and liver. Other characteristics such as normal or intact hooks and calcareous corpuscules might be observed in live PSCs but not in dead PSCs.

$$\begin{matrix} \%viability=\frac{N^{\circ}live PSCs}{N^{\circ}Total PSCs}x100 \\ \end{matrix}$$

**Data analysis**

The Kruskall-Wallis test will be used to estimate the difference in the number of cysts between treatment groups. Analysis of Variance (ANOVA) will be performed for cyst length and Bonferroni multiple-comparison test used to establish the pair-group difference. The chi-square test will be used to assess the association of fertility and degenerated cysts (calcified and purulent cysts included) with treatment groups. Logistic regression models will be used to estimate the odds of having degenerated or fertile cysts with treatment and organ as the independent variables with cluster in animal codes. Following previous field experience [2], we will define sheep as “cured” if all fertile cysts in the animal have no viable protoscoleces (0% viability); “improved” sheep will be those that have fertile cysts between 1% to 60% protoscolex viability; and “unchanged” sheep will be those whose fertile cysts had more than 60% protoscolex viability. For the purposes of this study, drug efficacy will be approached as the cured and/or improved sheep rates. For all the analyses, the significant differences will be set to less than 0.05. Percentages will be presented with their 95% confidence intervals.

**FIELD FORM FOR SHEEP (NOTE: USE ONE FORM PER ORGAN)**

**CODE: ……………. DATE ………………….. FACILITATOR NAME…………………………**

**ORGAN: LUNG DIAGNOSTIC: POSITIVE N° CYSTS: RL …… LL……. LIV ……..**

**LIVER NEGATIVE**

**OTHER PARASITES: Dyctiocaulus Coenurus C. tenuicollis F. liver Tysanosoma**

0=no fertile

1=fertile

| **ID** | **LENGTH**  **(mm)** | **LIQUID(1)** | | | | | **FERTIL** | **VIAB**  **%** | **APPEARANCE(2)** | | | | **NOTES** |
| --- | --- | --- | --- | --- | --- | --- | --- | --- | --- | --- | --- | --- | --- |
|  |  | **NO** | **TRA** | **YEL** | **PUR** | **BLO** |  |  | **NOR** | **CALC** | **DEG** | **HAEM** |  |
| **1** |  |  |  |  |  |  |  |  |  |  |  |  |  |
| **2** |  |  |  |  |  |  |  |  |  |  |  |  |  |
| **3** |  |  |  |  |  |  |  |  |  |  |  |  |  |
| **4** |  |  |  |  |  |  |  |  |  |  |  |  |  |
| **5** |  |  |  |  |  |  |  |  |  |  |  |  |  |
| **6** |  |  |  |  |  |  |  |  |  |  |  |  |  |
| **…** |  |  |  |  |  |  |  |  |  |  |  |  |  |
| **10** |  |  |  |  |  |  |  |  |  |  |  |  |  |

**^(1)^: NO=lack of liquid; TRA=transparent; YEL=yellowish; PUR=purulent; BLO=bloddy.**

**^(2)^: NOR=normal; CALC=calcified; DEG=degenerade; HAEM=haemorrhágic**

**REGISTRATION AND CODE TABLE**

**(IDENTIFICATION TABLE (“BLIND”) – ONLY FOR PERSON IN CHARGE)**

Assigned

Number for Blind Study

| **DATE** | **EAR TAG** | **GROUP** | **CODE** | **BLOOD SAMPLE** | | **WEIGHT CALCULATION** | |
| --- | --- | --- | --- | --- | --- | --- | --- |
|  |  |  |  | **YES** | **NO** | **W/ TAPE** | **WEIGHT** |
|  |  |  |  |  |  |  |  |
|  |  |  |  |  |  |  |  |
|  |  |  |  |  |  |  |  |
|  |  |  |  |  |  |  |  |
|  |  |  |  |  |  |  |  |
|  |  |  |  |  |  |  |  |
|  |  |  |  |  |  |  |  |
|  |  |  |  |  |  |  |  |
|  |  |  |  |  |  |  |  |
|  |  |  |  |  |  |  |  |
|  |  |  |  |  |  |  |  |
|  |  |  |  |  |  |  |  |
|  |  |  |  |  |  |  |  |
|  |  |  |  |  |  |  |  |
|  |  |  |  |  |  |  |  |

# References

1. Rosner, B., *Hypothesis Testing: Categorical Data*, in *Fundamentals of Biostatistics*. 2000, Duxbury Thomson Learning: Pacific Grove, CA. p. 355-424.

2. Dueger, E.L., P.L. Moro, and R.H. Gilman, *Oxfendazole Treatment of Sheep with Naturally Acquired Hydatid Disease.* Antimicrobial Agents and Chemotherapy, 1999. **43**(9): p. 2263-2267.

3. Blanton, R.E., et al., *Oxfendazole Treatment for Cystic Hydatid Disease in Naturally Infected Animals.* Antimicrobial Agents and Chemotherapy, 1998. **42**(3): p. 601-05.

4. Moro, P.L., et al., *Epidemiology of Echinococcus granulosus infection in the central Peruvian Andes.* Bull World Health Organ, 1997. **75**(6): p. 553-61.

5. Himonas, C., K. Antoniadou-Sotiriadou, and P. E., *Hydatidosis of food animals in Greece: prevalence of cysts containing viable protoscoleces.* J Helminthol, 1994. **68**(4): p. 311-3.
